# Lower Psychological Well-Being and Excessive Sexual Interest Predict Symptoms of Compulsive Use of Sexually Explicit Internet Material Among Adolescent Boys

**DOI:** 10.1007/s10964-015-0326-9

**Published:** 2015-07-25

**Authors:** Suzan M. Doornwaard, Regina J. J. M. van den Eijnden, Laura Baams, Ine Vanwesenbeeck, Tom F. M. ter Bogt

**Affiliations:** Department of Interdisciplinary Social Science, Utrecht Center for Child and Adolescent Studies, Utrecht University, Heidelberglaan 1, P.O. Box 80140, 3508 TC Utrecht, The Netherlands; Department of Developmental Psychology, Utrecht Center for Child and Adolescent Studies, Utrecht University, P.O. Box 80140, 3508 TC Utrecht, The Netherlands

**Keywords:** Adolescents, Compulsive use, Sexually explicit Internet material, Symptoms, Psychological well-being, Sexual interest

## Abstract

Although a growing body of literature addresses the effects of young people’s use of sexually explicit Internet material, research on the compulsive use of this type of online content among adolescents and its associated factors is largely lacking. This study investigated whether factors from three distinct psychosocial domains (i.e., psychological well-being, sexual interests/behaviors, and impulsive-psychopathic personality) predicted symptoms of compulsive use of sexually explicit Internet material among adolescent boys. Links between psychosocial factors and boys’ compulsive use symptoms were analyzed both cross-sectionally and longitudinally with compulsive use symptoms measured 6 months later (T_2_). Data were used from 331 Dutch boys (*M*_age_ = 15.16 years, range 11–17) who indicated that they used sexually explicit Internet material. The results from negative binomial regression analyses indicated that lower levels of global self-esteem and higher levels of excessive sexual interest concurrently predicted boys’ symptoms of compulsive use of sexually explicit Internet material. Longitudinally, higher levels of depressive feelings and, again, excessive sexual interest predicted relative increases in compulsive use symptoms 6 months later. Impulsive and psychopathic personality traits were not uniquely related to boys’ symptoms of compulsive use of sexually explicit Internet material. Our findings, while preliminary, suggest that both psychological well-being factors and sexual interests/behaviors are involved in the development of compulsive use of sexually explicit Internet material among adolescent boys. Such knowledge is important for prevention and intervention efforts that target the needs of specific problematic users of sexually explicit Internet material.

## Introduction

The proliferation of Internet access worldwide and the rapid development of Internet-enabled devices have changed the way young people encounter, consume, and distribute content of all kinds. One area of content that has received particular attention in this regard is sexually explicit Internet material (Wolak et al. 2007). Compared to other media, the Internet is a highly sexualized environment, characterized by an abundance and unprecedented variety of sexual materials (Peter and Valkenburg 2006). In addition, the Internet has several properties that make it a particularly attractive medium for consuming sexual content. For example, Cooper (1998) has described the Internet in terms of a *Triple A Engine* of accessibility, affordability, and anonymity. Moreover, Young’s (1999) *ACE model* highlights anonymity, convenience, and escape as highly appealing facets. These characteristics of the Internet can be positive; for example, they may facilitate the age-normative exploration of sexuality in adolescence (Wolak et al. 2007). On the other hand, the easy and anonymous access to all conceivable kinds of sexual content may leave users vulnerable for developing tendencies of compulsive use of sexually explicit Internet material or other problematic types of sex-related Internet use.

One group that may be particularly at risk for developing compulsive or problematic tendencies related to the use of sexually explicit Internet material are adolescents, who are going through a phase of increased sexual curiosity (Savin-Williams and Diamond 2004) in the context of almost unlimited and often unmonitored access to the Internet (Madden et al. 2013). Although the majority of youth using online sexual content do not develop compulsive tendencies, for those who do, their patterns of use may have significant and enduring consequences in many areas of their lives (Cooper et al. 2004; Sussman 2007). For example, there is evidence among adult diagnosed sex addicts that acting out sexual behavior may already begin in preadolescence or adolescence—often with an excessive interest in pornography (Cooper et al. 1999; Sussman 2007). Therefore, it is of critical importance to understand when and for whom use of sexually explicit Internet material may be particularly problematic. Yet, research on compulsive use of sexually explicit Internet material among adolescents and its associated factors is largely lacking. The goal of the current study is to address this gap in the literature by investigating the psychosocial factors that put male adolescent users of this type of online content at increased risk for developing symptoms of compulsive use.

### Compulsive Use of Sexually Explicit Internet Material

One of the reasons for the scarcity of studies on compulsive use of sexually explicit Internet material—or overlapping phenomena such as problematic/pathological sex-related Internet use or online pornography addiction—among adolescents might be the lack of consistent conceptualizations, definitions, and classifications of the phenomenon. For example, the frequency of using sexually explicit Internet material alone may not be sufficient to determine when the behavior is adaptive or problematic, as some may use sexually explicit material regularly without experiencing any discomfort, while others consider their use problematic even if it is minimal from an absolute time perspective (Davis 2001; Grubbs et al. 2015)—and these subjective experiences also likely vary with age. Furthermore, it is unclear whether compulsive use of sexually explicit Internet material is a manifestation of Internet addiction, a technological variant of hypersexual behavior, or a disorder on its own (Griffith 2004; Ross et al. 2012). Despite this lack of consensus on definitions and classifications, researchers and clinicians generally agree on several core criteria of compulsive use of sexually explicit Internet material, which are comparable with criteria for other addictive disorders (e.g., gambling disorder). These include a perceived lack of control over one’s use or the inability to stop despite negative adverse consequences; persistent thoughts about or a preoccupation with using sexually explicit Internet material; and severe adverse consequences as a result of one’s use, such as damaged relationships, school or work problems (Delmonico and Griffin 2008; Grubbs et al. 2015; Ross et al. 2012; Twohig et al. 2009). Additional core criteria described in the literature are the use of sexually explicit Internet material to cope with or escape from negative feelings and the experience of unpleasant emotions when use is impossible (Delmonico and Griffin 2008; Meerkerk et al. 2009).

### Factors Associated with Compulsive Use of Sexually Explicit Internet Material

Parallel to the debate about its conceptualization is the study of factors associated with the development of compulsive use of sexually explicit Internet material. Prior research has linked the problematic use of online sexual content to a number of other risk factors and comorbid conditions, including depression, anxiety, and low self-esteem (Cooper et al. 1999, 2004; Delmonico and Griffin 2008; Grubbs et al. 2015), social isolation (Boies et al. 2004; Delmonico and Griffin 2008), sexual compulsiveness (Cooper et al. 1999, 2004; Delmonico and Griffin 2008; Grubbs et al. 2015), and antisocial personality traits (Bogaert 2001; Delmonico and Griffin 2008). Given this wide range of associated psychosocial factors, it is conceivable that the population of compulsive users of sexually explicit Internet material is not a homogenous group, but instead comprises distinct subtypes of users characterized by different underlying states or traits (Cooper et al. 1999; Nower and Blaszczynski 2004). Cooper et al. (1999) have addressed this issue in their study on sex-related Internet use, in which they describe different subtypes of Internet users who are at similar increased risk for developing pathological tendencies related to their online sexual behavior, but differ with respect to the factors involved in the progression from recreational to problematic sex-related Internet use. Specifically, the *at*-*risk* subtype consists of individuals characterized by poor psychological well-being, who have a tendency to engage in online sexual behavior in response to depressive or anxious feelings (i.e., the depressive type) or stressful situations (i.e., the stress-reactive type; Cooper et al. 1999, 2004). According to this perspective, adolescents would use sexually explicit Internet material as a potential coping mechanism; as a temporary escape, distraction, or way of relieving stress or negative affective states. What further characterizes individuals in the at-risk subtype is that they often have no history of sexual compulsivity, but may be more vulnerable to developing tendencies of sexual compulsiveness due to the convenient facets of the Internet. This is in contrast to Cooper et al.’s (1999) *sexually compulsive* subtype, which consists of individuals with past or present problems with sexual matters and for whom the Internet is merely an effective tool to cater to their persistent sexual needs (Cooper et al. 1999, 2004). According to this perspective, adolescents who show compulsive sexual tendencies offline are likely to replicate and amplify these tendencies online with their use of sexually explicit Internet material. As such, the sexually compulsive subtype corresponds with conceptualizations of compulsive use of sexually explicit Internet material as a technological variant of hypersexual behavior (e.g., Grubbs et al. 2015). It is important, however, to consider the concept of sexual compulsiveness within a developmental context. For adolescents, who are in the process of discovering and exploring sexuality, sexual “compulsiveness” may be a qualitatively different phenomenon, expressed in terms of an above average, excessive interest in sexual matters (sometimes referred to as sexual preoccupation) and earlier or more experience with sexual behavior, rather than with pathological or hypersexual behavior.

Like Cooper et al. (1999), Nower and Blaszczynski (2004) distinguished various subtypes of youth pathological gamblers. Although gambling and using sexually explicit Internet material are obviously different behaviors, the literature suggests that there is an overlap in both the core criteria of and the factors associated with pathological gambling and compulsive use of sexually explicit Internet material (e.g., Ross et al. 2012). Similar to Cooper et al. (1999), Nower and Blaszczynski (2004) describe in their Pathways Model of pathological gambling an at-risk subtype (labeled *emotionally*-*vulnerable*), which consists of individuals suffering from depression, anxiety, and low self-esteem, and for whom gambling functions as a means of coping with their negative feelings (Gupta et al. 2013). However, they also describe a different subtype of youth gamblers, labeled *antisocial*-*impulsivist*, whose members are mainly distinguished by features such as impulsivity, sensation-seeking, and psychopathic personality traits. It is thought that individuals in this subtype engage in gambling to achieve arousal and stimulation (Gupta et al. 2013; Nower and Blaszczynski 2004). Although Cooper et al. (1999) did not distinguish an antisocial-impulsivist subtype of sex-related Internet users, personality traits such as sensation-seeking have been associated with sexually explicit media use among adult as well as adolescent males (Peter and Valkenburg 2011) and among women (Vanwesenbeeck 2001). Moreover, Bogaert (2001) found that aggressive/antisocial tendencies were predictive of men’s preference for violent sexual media content.

At present, no studies have assessed whether these distinct psychosocial domains (i.e., psychological well-being, sexual interests/behaviors, and impulsive-psychopathic personality) are associated with the development of compulsive use of sexually explicit Internet material in adolescents.

## The Present Study

The goal of the present study was to examine, both concurrently and prospectively, how factors within the domains of (1) psychological well-being (i.e., depression, global self-esteem), (2) sexual interests/behaviors (i.e., excessive sexual interest, experience with sexual behavior), and (3) impulsive and psychopathic personality (i.e., impulsivity, affective and interpersonal psychopathic traits) are associated with symptoms of compulsive use of sexually explicit Internet material among adolescent boys. Our study does not aim to group diagnosed compulsive users of sexually explicit Internet material into distinct etiological subtypes, but rather seeks to identify the psychosocial factors that put male adolescent users of this material at increased risk for progressing to problematic use. Based on prior findings among sex-related Internet users (Cooper et al. 1999) and assumptions of the Pathways Model (Nower and Blaszczynski 2004), we expected that factors from the different domains (i.e., psychological well-being, sexual interests/behaviors, and impulsive-psychopathic personality) would be uniquely related to boys’ tendencies of compulsive use of sexually explicit Internet material. Specifically, we hypothesized that lower levels of psychological well-being (i.e., higher levels of depression and lower levels of global self-esteem), higher levels of sexual interests and behaviors, and higher levels of impulsive and psychopathic personality traits would predict higher scores on boys’ symptoms of compulsive use of sexually explicit Internet material.

## Method

### Participants

Data for this study were collected as part of Project STARS (Studies on Trajectories of Adolescent Relationships and Sexuality), a larger longitudinal research project on romantic and sexual development of Dutch adolescents. Prior to the first measurement, both adolescents and their parents received letters, brochures, and flyers describing the aims of the study and the possibility to decline or end participation at any time. Parents could return signed forms indicating that their child was not allowed to take part in the study. Adolescents with passive informed parental consent were ensured at each measurement occasion that participation was voluntary and that they could return to their classroom if they did not wish to take part in the study. For a full description of the longitudinal sample and study procedures, see Doornwaard et al. (2015). Study procedures were approved by the ethics board of the Faculty of Social and Behavioural Sciences of Utrecht University. For the present study, we selected data from the final two measurement waves (in the original project T_3_ and T_4_; in the current study T_1_ and T_2_, respectively) as our youngest participants did not complete all investigated concepts at earlier waves. We aimed to predict boys’^1^ symptoms of compulsive use of sexually explicit Internet material at both time-points; first cross-sectionally at T_1_ and subsequently longitudinally with symptoms of compulsive use measured 6 months later (T_2_).

Three hundred forty-six boys who indicated they used sexually explicit Internet material at T_1_ were eligible for the cross-sectional analyses. Of those, 15 were excluded due to unreliable data, leaving a total of 331 participants. The average age of this sample was 15.16 years (*SD* = 1.31; range 11–17). Most boys had a Dutch (i.e., self and both parents born in the Netherlands; 78.2 %) or Western (i.e., self or a parent born in Europe, the United States, Canada, Australia, or New-Zealand; 12.1 %) background; the remaining 9.7 % had a non-Western background (i.e., self or a parent born in an African, Middle Eastern, Asian, or South American country). Boys were enrolled in different educational tracks, with 50.0 % in vocational programs and 50.0 % in college/university preparatory programs. The majority of boys reported being heterosexual (97.9 %) and single (89.1 %).

Of the 331 boys in the cross-sectional analyses, 251 reported to use sexually explicit Internet material at T_2_ as well; they were therefore included in the longitudinal analyses. Of the 80 excluded participants, 56 (70 %) were excluded because they did not complete the T_2_ questionnaire and 24 (30 %) were excluded because they reported no use of sexually explicit Internet material at T_2_. Compared to participants who were retained in the longitudinal sample, participants who were excluded were somewhat older at T_1_, *t*(329) = 3.42, *p* < .001, and more often had a non-Western background, *χ*^2^(1, *N* = 331) = 7.41, *p* = .006.

### Measures

#### Compulsive Use of Sexually Explicit Internet Material

Compulsive use of sexually explicit Internet was measured with six items from the Compulsive Internet Use Scale (Meerkerk et al. 2009), which were modified to assess symptoms of compulsive searching for/viewing of pornography on the Internet, instead of general compulsive Internet use symptoms (Table 1). The six items reflect the five core criteria for compulsive use of sexually explicit Internet material: lack of control over one’s use (item 1); preoccupation with use (items 2 and 4); adverse consequences as a result of one’s use (item 3); experience of unpleasant emotions when use is impossible (item 5); and use to cope with or escape from negative feelings (item 6). Adolescents rated, on a 6-point scale (0 = *Never*, 1 = *Rarely*, 2 = *Sometimes*, 3 = *Regularly*, 4 = *Often*, 5 = *Very often*), how frequently they had experienced each of the symptoms when searching for and viewing porn on the Internet. The items were summed, resulting in a compulsive use of sexually explicit Internet material scale ranging from 0 (*Experienced no symptoms*) to 30 (*Experienced all six symptoms very often*); Cronbach’s α for this measure was .85 at T_1_ and .83 at T_2_.

**Table 1:** Adapted items to assess compulsive SEIM use and occurrence of symptoms among 331 SEIM using adolescent boys

Item	Never *N* (*%*)	Rarely *N* (*%*)	Sometimes *N* (*%*)	Regularly *N* (*%*)	Often *N* (*%*)	Very often *N* (*%*)
1. How often do you find it difficult to stop searching for/viewing porn on the Internet?	266 (80.4)	38 (11.5)	16 (4.8)	6 (1.8)	2 (0.6)	3 (0.9)
2. How often do you prefer to search for/view porn on the Internet instead of spending time with others (e.g., friends or parents)?	276 (83.4)	39 (11.8)	10 (3.0)	5 (1.5)	1 (0.3)	0 (0.0)
3. How often are you short of sleep because of searching for/viewing porn on the Internet?	294 (88.8)	23 (7.0)	8 (2.4)	5 (1.5)	0 (0.0)	1 (0.3)
4. How often do you look forward to the next time you can search for/view porn on the Internet?	223 (67.4)	71 (21.4)	25 (7.6)	7 (2.1)	2 (0.6)	3 (0.9)
5. How often do you feel down or irritated when you are not able to search for/view porn on the Internet?	291 (87.9)	26 (7.9)	8 (2.4)	3 (0.9)	1 (0.3)	2 (0.6)
6. How often do you search for/view porn on the Internet because you are feeling down?	294 (88.8)	21 (6.4)	10 (3.0)	5 (1.5)	1 (0.3)	0 (0.0)

Items adapted from the Compulsive Internet Use Scale ([CIUS]; Meerkerk et al., 2009)

*SEIM* sexually explicit Internet material

#### Psychological Well-Being

*Depressive symptoms* were measured with six items from the Depressive Mood List (Kandel and Davies 1982). Adolescents rated on a 5-point scale (1 = *Never*, 5 = *Always*) how often they had experienced each of six negative feelings in the previous 6 months (e.g., “I felt too tired to do something”; α_T1_ = .85, α_T2_ = .83). *Global self*-*esteem* was assessed using an adapted version of the Global Self-Worth subscale of the Self-Perception Profile for Adolescents (Harter 1985, 2012; Straathof and Treffers 1989; Wichstrøm 1995). Adolescents rated on a 5-point scale (1 = *Completely untrue*, 5 = *Completely true*) how much each of five descriptions applied to them (e.g., “I am often disappointed in myself” [reversed]; α_T1_ = .78, α_T2_ = .75).

#### Sexual Interests/Behaviors

*Excessive sexual interest* was measured with four items from the Sexual-Preoccupation subscale of Snell and Papini’s (1989) Sexuality Scale. Adolescents rated on a 6-point scale (1 = *Completely disagree*, 6 = *Completely agree*) the extent to which they agreed with each of the statements about their interest in sex (e.g., “I think about sex a great deal of the time”, “I probably think about sex more than other people”; α_T1_ = .89, α_T2_ = .94). To assess adolescents’ *experience with sexual behavior*, participants initially were asked two questions: “Have you ever French kissed somebody?” and “Have you ever had sex with another person? With sex we mean everything from touching or caressing to intercourse.” (0 = *No*, 1 = *Yes*). Those who indicated *Yes* on the second question received follow-up questions about their experience with four different sexual behaviors: (a) naked touching or caressing, (b) performing or receiving manual sex, (c) performing or receiving oral sex, and (d) vaginal or anal intercourse (0 = *No*, 1 = *Yes*). The kissing and four sexual behavior items were combined into one variable measuring the level of adolescents’ experience with sexual behavior, ranging from 0 = *Inexperienced with all five behaviors* to 5 = *Experience with five behaviors* (α_T1_ = .85, α_T2_ = .86).

#### Impulsive and Psychopathic Personality

Adolescents’ level of *impulsivity* was measured with five items from the Eysenck Impulsiveness Scale (Eysenck and Eysenck 1978; Vitaro et al. 1997). Adolescents rated on a 5-point scale (1 = *Completely disagree,* 5 = *Completely agree*) the extent to which they agreed with each statement about themselves (e.g., “I usually do and say things without thinking about it”; α_T1_ = .86, α_T2_ = .85). *Affective psychopathic traits* were measured with the *callous*-*unemotional* dimension of the Youth Psychopathic Traits Inventory-Short Version (Andershed et al. 2007; Hillege et al. 2010; Van Baardewijk et al. 2010). This dimension consists of six statements reflecting remorseless, unemotional, or callousness beliefs (e.g., “If other people have problems, it usually is their own fault and therefore you should not help them”; α_T1_ = .77, α_T2_ = .76). Adolescents were asked to indicate on a 4-point scale (1 = *Does not apply at all*, 4 = *Applies very well*) how they generally think or feel about each statement, not just at that moment. The instructions further stressed that there were no right or wrong answers. *Interpersonal psychopathic traits* were assessed with the *grandiose*-*manipulative* dimension of the Youth Psychopathic Traits Inventory-Short Version (Andershed et al. 2007; Hillege et al. 2010; Van Baardewijk et al. 2010). With the same instructions as the callous-unemotional items, adolescents rated six statements reflecting dishonest charm, manipulative, and grandiose beliefs and behaviors (e.g., “I have the ability to con people by using my charm and smile”; α_T1_ = .88, α_T2_ = .89).

### Data Analyses

Descriptive statistics and correlations among the variables of interest were obtained. To examine the predictive role of factors in the three psychosocial domains (i.e., psychological well-being, sexual interests/behaviors, impulsive-psychopathic personality) in the development of symptoms of compulsive use of sexually explicit Internet material among adolescent boys, we performed negative binomial regression analyses. As is often the case with disorders and addictions, the distribution of our dependent variable, symptoms of compulsive use of sexually explicit Internet material, was dominated by zero values (53.8 % at T_1_ and 47.8 % at T_2_) while increasing values declined in frequency. As a consequence, this count variable was “over-dispersed”; that is, its variance was greater than its mean, which may result in underestimation of standard errors when normal Poisson regressions for count data are used. Negative binomial models correct for this over-dispersion and therefore produce more reliable estimates (Cameron and Trivedi 1998).

Model estimations were similar for the cross-sectional and longitudinal analyses, with the only exception being that the cross-sectional models included symptoms of compulsive use of sexually explicit Internet material at T_1_ as the dependent variable, whereas the longitudinal models included compulsive use symptoms at T_2_ as the dependent variable and compulsive use symptoms at T_1_ as a control variable. First, a regression model was estimated with the T_1_ psychological well-being predictors (depression, global self-esteem); second, a model was estimated with the T_1_ sexual interests/behaviors predictors (excessive sexual interest, experience with sexual behavior); and third, a model was estimated with the T_1_ impulsive and psychopathic personality predictors (impulsivity, affective and interpersonal psychopathic traits). Finally, to assess the unique role of the three domains in predicting boys’ symptoms of compulsive use of sexually explicit Internet material, a model was estimated with the significant predictors from the previous three models. All models included age at T_1_ as a control variable. Maximum likelihood robust estimation was used to estimate models. Analyses were conducted in Mplus (Version 7.3; Muthén and Muthén 2014).

## Results

Table 1 presents the occurrence of the six symptoms of compulsive use of sexually explicit Internet material in the cross-sectional sample of 331 boys. As expected, most male adolescent users of sexually explicit Internet material did not report any compulsive tendencies related to their use. Yet, symptoms of compulsive use were experienced at least “sometimes” by 4.2–11.2 % of the sample. The average score on the combined measure of compulsive use of sexually explicit Internet material at T_1_ was 1.63 (*SD* = 3.15) with a minimum of 0 and a maximum of 24 (median = 0); the average score at T_2_ was 1.98 (*SD* = 3.29) with a minimum of 0 and a maximum of 19 (median = 1). Table 2 shows correlations (cross-sectional and longitudinal) among the variables of interest. Higher levels of impulsivity and excessive sexual interest and lower levels of global self-esteem were cross-sectionally associated with higher scores on boys’ symptoms of compulsive use of sexually explicit Internet material. Longitudinally, higher levels of depression, affective psychopathic traits, and excessive sexual interest, and lower levels of global self-esteem were associated with higher scores on compulsive use of sexually explicit Internet material 6 months later (see Table 2).

**Table 2:** Descriptive statistics and correlations between boys’ compulsive SEIM use and measures of psychological well-being, sexual interests/behaviors, and impulsive-psychopathic personality

	*M* (*SD*) T_1_ (*N* = 331)	*M* (*SD*) T_2_ (*N* = 251)	1	2	3	4	5	6	7	8
1. Compulsive SEIM use	1.63 (3.15)	1.98 (3.29)	–	.20**	−.15*	.27***	−.07	.09	.16*	.05
2. Depression	2.26 (0.71)	–	.06	–	−.49***	.15*	−.02	.16*	.15	.03
3. Global self-esteem	4.18 (0.69)	–	−.17***	−.45***	–	−.28***	.07	−.07	−.13*	−.01
4. Excessive sexual interest	2.01 (0.84)	–	.32***	.12	−.23***	–	.07	.17*	.11	.15
5. Experience with sexual behavior	1.30 (1.57)	–	−.01	.04	.08	.09	–	.18*	.10	.16**
6. Impulsivity	2.67 (0.81)	–	.10*	.18**	−.10	.18**	.15**	–	.19**	.40***
7. Affective psychopathy	1.76 (0.56)	–	.11	.21**	−.11	.12	.12	.21***	–	.26***
8. Interpersonal psychopathy	1.89 (0.72)	–	.13	.05	.01	.10	.17**	.33***	.27***	–

Cross-sectional (T_1_) correlations are presented below the diagonal (*N* = 331); longitudinal correlations (T_1_ predictors with T_2_ compulsive SEIM use) are presented above the diagonal (*N* = 251)

*SEIM* sexually explicit Internet material

* *p* < .05; ** *p* < .01; *** *p* < .001 (two-tailed)

To assess the unique importance of these factors in predicting boys’ symptoms of compulsive use of sexually explicit Internet material, negative binomial regression analyses were conducted. Table 3 shows the results of the cross-sectional (left column) and longitudinal (right column) models. Cross-sectionally, factors within two domains emerged as significant predictors of symptoms of compulsive use of sexually explicit Internet material. Specifically, in the psychological well-being model (Model 1), global self-esteem negatively predicted compulsive use symptoms, indicating that boys with relatively lower levels of global self-esteem are at increased risk for the development of problematic use of sexually explicit Internet material. Moreover, in the sexual interests/behaviors model (Model 2), excessive sexual interest positively predicted compulsive use symptoms. No factors in the impulsive-psychopathic personality model (Model 3) significantly predicted symptoms of compulsive use of sexually explicit Internet material. When the significant factors from the psychological well-being and sexual interests/behaviors domains were considered jointly in a fourth model, global self-esteem and excessive sexual interest both remained significant and unique predictors of boys’ compulsive use symptoms (see Table 3; left column).

**Table 3:** Results from negative binomial regression models predicting boys’ compulsive SEIM use at T_1_ (cross-sectionally; left column) and T_2_ (longitudinally; right column)

	Cross-sectional models (*N* = 331)		Longitudinal models (*N* = 251)	
	*B* (*SE*)	RR (95 % CI)	*B* (*SE*)	RR (95 % CI)
*Model 1: Psychological well-being T*_*1*_				
Compulsive SEIM use T_1_^a^	–	–	0.18 (.03)***	1.19 (1.13, 1.26)
Age	0.00 (.10)	1.00 (0.81, 1.20)	−0.11 (.06)	0.90 (0.79, 1.01)
Depression	−0.05 (.18)	0.95 (0.61, 1.30)	0.53 (.21)*	1.70 (1.00, 2.40)
Global self-esteem	−0.59 (.16)***	0.55 (0.38, 0.73)	0.13 (.15)	1.14 (0.80, 1.48)
*Model 2: Sexual interests/behaviors T*_*1*_				
Compulsive SEIM use T_1_^a^	–	–	0.15 (.03)***	1.16 (1.10, 1.22)
Age	−0.10 (.09)	0.99 (0.81, 1.17)	−0.08 (.07)	0.92 (0.79, 1.05)
Excessive sexual interest	0.65 (.13)***	1.92 (1.45, 2.39)	0.29 (.12)*	1.34 (1.04, 1.64)
Experience with sexual behavior	0.03 (.07)	0.97 (0.84, 1.11)	−0.04 (.09)	0.96 (0.78, 1.13)
*Model 3: Impulsive-psychopathic personality T*_*1*_				
Compulsive SEIM use T_1_^a^	–	–	0.17 (.03)***	1.19 (1.12, 1.26)
Age	−0.04 (.08)	0.96 (0.81, 1.12)	−0.08 (.07)	0.92 (0.80, 1.04)
Impulsivity	0.14 (.13)	1.15 (0.87, 1.43)	0.08 (.11)	1.08 (0.84, 1.33)
Affective psychopathy	0.20 (.19)	1.23 (0.78, 1.68)	0.29 (.19)	1.33 (0.84, 1.83)
Interpersonal psychopathy	0.17 (.16)	1.19 (0.81, 1.57)	−0.19 (.15)	0.83 (0.59, 1.06)
*Model 4: Significant predictors T*_1_				
Compulsive SEIM use T_1_^a^	–	–	0.15 (.03)***	1.16 (1.10, 1.22)
Age	−0.00 (.08)	1.00 (0.85, 1.15)	−0.10 (.06)	0.90 (0.79, 1.01)
Depression^b^	–	–	0.45 (.18)*	1.57 (1.02, 2.12)
Global self-esteem^c^	−0.44 (.17)**	0.65 (0.43, 0.86)	–	–
Excessive sexual interest	0.60 (.13)***	1.83 (1.38, 2.28)	0.26 (.14)	1.30 (0.95, 1.65)

*SEIM* sexually explicit Internet material, *RR* rate ratio

* *p* < .05; ** *p* < .01; *** *p* < .001 (two-tailed)

^a^Variable only included in longitudinal analyses

^b^Variable only significant in longitudinal analyses; therefore not included in final cross-sectional model

^c^Variable only significant in cross-sectional analyses; therefore not included in final longitudinal model

By adjusting for baseline measures of symptoms, longitudinal analyses enable the identification of risk factors that predict relative increases in boys’ symptoms of compulsive use of sexually explicit Internet material over time. In the psychological well-being model, depression predicted relatively higher scores on compulsive use symptoms 6 months later at T_2_. In addition, in the sexuality model, excessive sexual interest predicted relatively higher scores on compulsive use symptoms at T_2_. Impulsive-psychopathic personality traits did not longitudinally predict symptoms of compulsive use of sexually explicit Internet material. When depression and excessive sexual interest were considered jointly (Model 4), only depression remained a significant predictor of relatively higher scores on symptoms of compulsive use of sexually explicit Internet material at T_2_ (see Table 3; right column).

## Discussion

Although research on the effects of young people’s use of sexually explicit Internet material has grown steadily over the past years, knowledge on compulsive use of this type of online content among adolescents is largely lacking. Researchers and clinicians have pointed out that compulsive sex-related online behavior during adolescence may have serious and enduring implications throughout development. For example, many adult diagnosed sex addicts have reported that their acting out sexual behavior started in preadolescence or adolescence—often with an excessive interest in pornography (Cooper et al. 1999; Sussman 2007). Therefore, identifying the factors that are associated with a heightened vulnerability for developing tendencies of compulsive use of sexually explicit Internet material during adolescence is vital. The aim of this study was to investigate how factors from three distinct psychosocial domains (i.e., psychological well-being, sexual interests/behaviors, and impulsive-psychopathic personality) predicted symptoms of compulsive use of sexually explicit Internet material among adolescent boys.

### Psychosocial Factors Predicting Boys’ Symptoms of Compulsive Use of Sexually Explicit Internet Material

As expected, most users of sexually explicit Internet material in our sample of Dutch male adolescents did not report any compulsive tendencies related to their use. Nonetheless, a small group of boys (i.e., between 4.2 and 11.2 %) did experience compulsive use symptoms on an occasional basis. Results of our cross-sectional analyses showed that lower levels of global self-esteem and higher levels of excessive sexual interest predicted boys’ symptoms of compulsive use of sexually explicit Internet material. Furthermore, longitudinal analyses indicated that higher levels of depressive feelings and, again, excessive sexual interest predicted relatively higher scores on boys’ symptoms of compulsive use of sexually explicit Internet material 6 months later, with the former being the most consistent predictor. Interestingly, global self-esteem and depression appeared as significant predictors in separate analyses. It should be noted, though, that these factors were strongly interrelated. Therefore, the non-significance of global self-esteem in the longitudinal analyses and the non-significance of depression in the concurrent analyses do not imply that these factors are unimportant predictors. Rather, low global self-esteem and depressive feelings may both be manifestations of a deeper rooted negative affective state. Impulsive and psychopathic personality traits, which were significantly associated with compulsive use symptoms in bivariate analyses, were no unique predictors when included in the multivariate regression models.

These findings support notions from the literature, as well as the hypothesis of this study, that different psychosocial domains are involved in the development of compulsive use of sexually explicit Internet material (e.g., Cooper et al. 1999, 2004; Nower and Blaszczynski 2004). First, consistent with assumptions of the Pathways Model (Nower and Blaszczynski 2004) and findings among adult cybersex users (Cooper et al. 2004), our results demonstrate that adolescent boys characterized by lower psychological well-being are at increased risk for progressing to problematic use of sexually explicit Internet material. Prior studies have repeatedly linked frequent and/or compulsive use of (online) sexual content to psychological distress (e.g., Cooper et al. 2004; Delmonico and Griffin 2008; Grubbs et al. 2015; Sussman 2007). Although their designs precluded an examination of the causal direction of this relation, many of these studies have suggested that individuals suffering from poor psychological well-being may use online sexual content as a coping mechanism or a way of relieving their dysphoria. Our longitudinal analyses offer preliminary support for this idea by showing that higher levels of depression predicted relative increases in boys’ symptoms of compulsive use of sexually explicit Internet material 6 months later. This finding may indicate that boys experiencing depressive or anxious feelings turn to this material in an attempt to escape from or diminish their negative affective states; yet, in doing so, they develop additional problems. However, it is also possible that poor psychological well-being and compulsive use of sexually explicit Internet material are reciprocally related and reinforce each other over time. For example, compulsive users of sexually explicit Internet material may experience feelings of depression and diminished self-esteem as they notice the adverse consequences of their use, which, in turn, may lead to further increases in their use to cope with their emotional distress (Cooper et al. 1999). More longitudinal research using cross-lagged panel designs is needed to establish the direction of these relations and inform treatment programs for both emotional problems and compulsive sex-related Internet use.

Second, our results indicate that adolescent boys with excessive sexual interest are at increased risk for developing tendencies of compulsive use of sexually explicit Internet material. The conceptualization of compulsive use of sexually explicit Internet material as a technological variant of hypersexual behavior or sex addiction has been a topic of debate (Griffith 2004; Ross et al. 2012), and although some studies indeed showed that self-reported high sexual desire was the strongest predictor of problematic use of such material (Svedin et al. 2011; Twohig et al. 2009), others found no relationship between this phenomenon and sexuality-related variables (Ross et al. 2012). These inconsistent findings may be explained by Cooper et al.’s (1999) theoretical distinction between emotionally-vulnerable (i.e., often no history of sexual problems) and sexually compulsive (i.e., characterized by sexual problems/acting out sexual behavior) subtypes of Internet users. That is, excessive sexual interest may be the underlying problem for some, but not all compulsive users of sexually explicit Internet material. Although the analytical design of our data did not allow us to empirically identify different subtypes, our results partly support the distinction proposed by Cooper et al. (1999), by showing that both global self-esteem and excessive sexual interest remained significant concurrent predictors of symptoms of compulsive use of sexually explicit Internet material when jointly considered. It should be noted that in the longitudinal analyses the statistical significance of excessive sexual interest did disappear when modeled together with depression; however, this finding may likely be explained by the fact that prior symptoms of compulsive use of sexually explicit Internet material (at T_1_) had already explained a considerable proportion of variance, leaving a smaller amount of variance to be explained by the psychosocial factors. Longitudinal and person-centered approaches, such as latent class growth analysis, would be useful to elucidate the processes involved in the development of compulsive use of sexually explicit Internet material in potentially distinct subtypes of young users. Knowledge on different subtypes of young compulsive users and their unique characteristics and etiology may guide health professionals by improving the early identification of at-risk youth and the development of tailored prevention and intervention efforts.

Our results provide little empirical support for the role of impulsive and psychopathic personality traits in the development of compulsive use of sexually explicit Internet material among adolescent boys. A possible explanation for the lack of findings with respect to this domain is that using sexually explicit Internet material—generally a solitary behavior that occurs in privacy behind a screen—has relatively few immediate and concrete effects, such as monetary gains or losses (e.g., as a consequence of gambling), intoxication (e.g., as a consequence of substance use), or status gains (e.g., in peer contexts). As such, though sexually stimulating, sexually explicit Internet material may not offer the type of sensation or excitement that individuals high in impulsivity may specifically pursue. Instead, youth high in impulsivity or psychopathy may be more likely to seek opportunities to engage in offline sexual behavior with more possibilities for immediate gratification; an idea substantiated by our data showing significant positive associations between impulsivity and interpersonal psychopathic traits and boys’ experience with sexual behavior. In other words, it may be that Nower and Blaszczynski’s (2004) antisocial-impulsivist pathway is one specific to “high gain/high loss” behaviors such as gambling, and does not apply to male adolescents’ use of sexually explicit Internet material.

The attempts in this study to elucidate the psychosocial factors involved in the development of compulsive use of sexually explicit Internet material among adolescent boys are preliminary, and the results must be interpreted with some caution. Our study examined the predictors of *symptoms* of compulsive use of sexually explicit Internet material, rather than the characteristics of diagnosed compulsive users. It is possible that those with a full diagnosis are characterized by a different psychosocial profile. Moreover, we agree with other researchers (e.g., Sussman 2007) that it is not always unequivocally clear when adolescents’ use of sexually explicit Internet material should be considered compulsive or problematic, and when it should not. Given their rapidly changing hormonal levels and accompanying increases in sexual interest and exploration (Savin-Williams and Diamond 2004), experiences such as looking forward to the next time one can use sexually explicit Internet material, or finding it difficult to stop using such material, may be considered as being typical of the adolescent phase rather than symptoms of compulsive behavior (Sussman 2007). On the other hand, sexually explicit Internet material that is being used to escape negative affective states, or use of sexually explicit Internet material resulting in adverse consequences, may be viewed as causes for concern during any stage of development. Moreover, even when the use of sexually explicit Internet material is not compulsive, it may nonetheless affect a range of sexual attitudes, emotions, and behaviors—particularly among adolescents who are in the process of exploring and developing their sexual self (for a review, see Owens et al. 2012). As such, our results can be considered an important first step toward understanding the compulsive use of sexually explicit Internet material among adolescent boys, and may form a starting point for more comprehensive research into the phenomenon.

### Limitations

Some limitations of this study warrant discussion. First, our study only examined short-term relationships (i.e., concurrent associations and associations over a 6-month interval) between psychosocial factors and boys’ symptoms of compulsive use of sexually explicit Internet material. It is therefore not clear whether psychological well-being and excessive sexual interest form risk factors for compulsive use of sexually explicit Internet material later in adolescence or adulthood, or whether the relationships found in this study diminish as adolescents mature. Longitudinal research over longer time periods is needed to elucidate the stability of compulsive use of sexually explicit Internet material, as well as the role of distinct psychosocial domains in the onset and maintenance of compulsive use tendencies. Such studies should also look into the effects that compulsive use of sexually explicit Internet material may have on later psychosocial functioning. Second, this study utilized self-report measures, which may be subject to response bias. Although self-report is still the most common method to collect data on sexuality, it is well-documented that adolescents may underreport their sexual interests and (online) behaviors, due to fear of embarrassment, disapproval, or social sanctions (Brener et al. 2003). Third, our results are based on a convenience sample in the Netherlands that was recruited through schools. It may be that those youth suffering most from tendencies of compulsive use of sexually explicit Internet material were underrepresented in our sample, due to their higher likelihood of having school problems and/or other psychopathology in addition to their compulsive use of online sexual content (Sussman 2007). Hence, the extent to which our results can be generalized to other populations of adolescents requires further investigation. Future studies should also investigate tendencies of compulsive use of sexually explicit Internet material and its associated psychosocial factors among adolescent girls, which was not possible in our study because of girls’ low self-reported use of this material.

## Conclusion

The powerful and convenient facets of the Internet make the consumption of sexual materials easier than ever before; yet at the same time they may leave particularly adolescents vulnerable for developing problematic or compulsive tendencies related to the use of such materials. This study offered an important contribution to this relatively understudied phenomenon in adolescence, by showing that both lower psychological well-being and excessive sexual interest predict boys’ symptoms of compulsive use of sexually explicit Internet material. Identifying the psychosocial domains and factors that are uniquely related to tendencies of compulsive use of sexually explicit Internet material among adolescents is a critical first step in the development of more efficient screening and treatment protocols that target the needs of specific problematic users of this material. Knowledge on risk factors may also increase awareness among parents and teachers, stimulate open communication between them and adolescents about their Internet use and affective states, and improve the early signaling of problems. At the same time, more prospective and person-centered research is needed to identify and refine etiologically distinct profiles of young compulsive users of sexually explicit Internet material that should form the basis for tailored prevention and intervention efforts.

## Footnotes

1 Half of the Project STARS longitudinal sample consisted of girls. However, due to their low self-reported use of sexually explicit Internet material, we were not able to investigate girls’ symptoms of compulsive use of sexually explicit Internet material in the current study.
